# When the goal is to generate a series of activities: A self-organized simulated robot arm

**DOI:** 10.1371/journal.pone.0217004

**Published:** 2019-06-19

**Authors:** Tim Koglin, Bulcsú Sándor, Claudius Gros

**Affiliations:** 1 Institute for Theoretical Physics, Goethe University Frankfurt, Frankfurt am Main, Germany; 2 Department of Physics, Babeș-Bolyai University, Cluj-Napoca, Romania; Consejo Nacional de Investigaciones Cientificas y Tecnicas, ARGENTINA

## Abstract

Behavior is characterized by sequences of goal oriented conducts, such as food uptake, socializing and resting. Classically, one would define for each task a corresponding satisfaction level, with the agent engaging, at a given time, in the activity having the lowest satisfaction level. Alternatively, one may consider that the agent follows the overarching objective to generate sequences of distinct activities. To achieve a balanced distribution of activities would then be the primary goal, and not to master a specific task. In this setting the agent would show two types of behaviors, task-oriented and task-searching phases, with the latter interseeding the former. We study the emergence of autonomous task switching for the case of a simulated robot arm. Grasping one of several moving objects corresponds in this setting to a specific activity. Overall, the arm should follow a given object temporarily and then move away, in order to search for a new target and reengage. We show that this behavior can be generated robustly when modeling the arm as an adaptive dynamical system. The dissipation function is in this approach time dependent. The arm is in a dissipative state when searching for a nearby object, dissipating energy on approach. Once close, the dissipation function starts to increase, with the eventual sign change implying that the arm will take up energy and wander off. The resulting explorative state ends when the dissipation function becomes again negative and the arm selects a new target. We believe that our approach may be generalized to generate self-organized sequences of activities in general.

## Introduction

Besides their industrial and practical applications, real and simulated robots are used increasingly to study the principles underlying embodied cognition [1] and locomotion [2], together with the self organization of critical sensorimotor states [3] and motor primitives [4]. Simulated robots may be considered in addition as proxies for cognitive and information processing agents [5].

It is well known that gaits and other regular muscle contractions, like breathing [6], are induced in many cases by central pattern generators [7, 8], even though it is currently controversial whether this is the case for biped locomotion [9], viz for human walking. Abstracting from animal models, one may ask conversely to which extent compliant locomotion may be generated via self-organizing principles [10], that is in the absence of top-down control in the form of a central pattern generator. One talks in this context of ‘embodiment’ [11], when part of the computation generating locomotion is carried out by the elasto-mechanical properties of the constituting body [12]. For quadruped robots with legs that are independently controlled by single non-linear phase oscillators [13], it has been shown that the limb-specific sensorimotor feedback derived form pressure sensors leads to self-organized interlimb communications, with emerging gaits that correspond to walking, trotting and galloping [14].

Self-organizing principles may be implemented within the sensorimotor loop [10], which is comprised of environment, body, actuator and sensory readings, with the latter being restricted in the pure case to propiosensation, viz to the internal state of the robot. The attractors self-stabilizing in the sensorimotor loop may then give rise to complex patterns of regular and of chaotic motion primitives [15], which can be selected in a second step using ‘kick control’ [16]. From a general perspective, kick control is an instance of a higher-level control mechanism exploiting the reduction in control complexity provided by morphologically computing robots [17, 18]. These approaches are hence different from other works where closed-loop policies are applied on the top of open-loop gait cycles [19, 20]. Alternatively, sequential switching between self-organizing behaviors in the combined phase space of the controller, body and environment can also be generated via self-exploration of the attractor landscape using an adaptive repelling potential [21].

Motor primitives and their generating guidelines are part of the basic constituents of a cognitive system [22]. Here we investigate whether self-organizing principles may be used also on a higher level. As a background we consider a setting where an agent has to follow a certain number of goals successively, with a typical example being that of an animal needing to forage, to watch out for predators, to rest and to socialize [23]. The agent is hence confronted with tasks that can be tackled only sequentially, a problem that may be cast into the framework of multi objective optimization [24], an approach which is however not taken in the present study. We examine instead to which extend a self-organized dynamical system may solve the time allocation problem implicitly.

**Fig 1:**
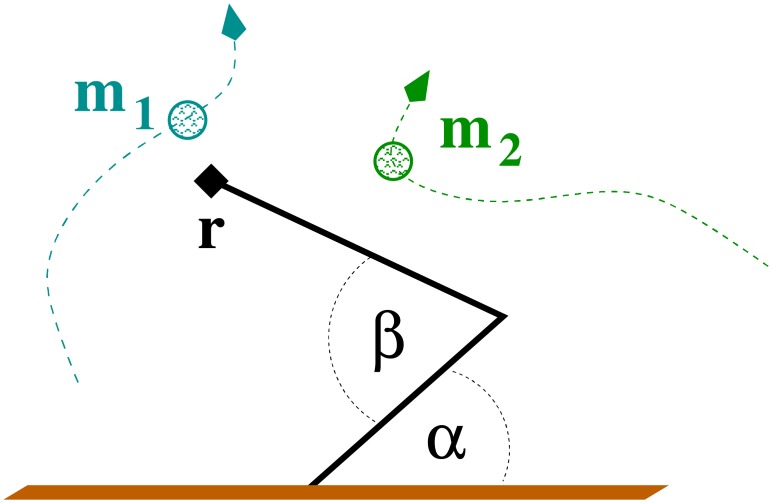
The simulated robot arm. The two angles *α* and *β* are actuated, with (5) governing the evolution of *α*. An equivalent dynamical system is in place for *β*. The arm has the task to catch one of the slowly moving objects **m**_*i*_, to follow it for a while, with **r** ≈ **m**_*i*_, and to switch autonomously to a distinct object.

As a basic protocol we consider an agent having to solve a series of indistinguishable tasks, with the agent being given by a simulated two-dimensional robot arm, as depicted in Fig 1. Within the reach of the arm there are a number of slowly moving objects the end actuator needs to reach and follow. Upon success, the self-organized dynamics of the arm should become ‘bored’ of the object, move away and search for a new one. We consider this protocol as a proxy for an agent showing a non-trivial sequence of behaviors generated not by top-down commands, but that emerges from underlying self-organizing principles.

## Materials and methods

The simulated robot arm sketched in Fig 1 has two degrees of freedom, the angles *α* and *β*, with the position **r** = (*r*_1_, *r*_2_) of the end effector, the hand, being given by

$$r_{1}=l_{1}\;\text{cos}\;\left(\alpha \right)-l_{2}\;\text{cos}\;\left(\beta -\alpha \right)$$ (1)

$$r_{2}=l_{1}\;\text{sin}\;\left(\alpha \right)+l_{2}\;\text{sin}\;\left(\beta -\alpha \right)\;,$$ (2)

where *l*_1_ and *l*_2_ are the respective arm lengths. We define a generalized potential *U* as

$$\begin{array}{c} U=U_{m}\prod_{i}T^{2}(R_{i}),\;\;R_{i}=\sqrt{(r-m_{i}{)}^{2}}, \end{array}$$ (3)

where *R*_*i*_ is the Euclidean distance between the position **m**_*i*_ of the *i*th target object and **r** = **r**(*α*, *β*). In (3) we used a squashing function *T*,

$$\begin{array}{c} T(z)=\kappa_{z}\;\text{tanh}\;(z/s_{z}),\;\;\frac{\partial T}{\partial \theta }=\frac{\kappa_{z}}{s_{z}}(1-\frac{T^{2}}{\kappa_{z}^{2}})\frac{\partial z}{\partial \theta }\;, \end{array}$$ (4)

which is characterized by a maximal value *κ*_*z*_ and a scale *s*_*z*_. We use *T*(*z*) throughout this study for the renormalization of several dynamical quantities, with the purpose to avoid exceedingly large forces or velocities. For the case of the distance we select a maximum value *κ*_*R*_ → 1, such that we have *T*(*R*_*i*_) = tanh(*R*_*i*_/*s*_*R*_), as entering (3). *U*_*m*_ is then the maximal value for the potential *U* = *U*(*α*, *β*).

### Robot arm dynamics

The dynamics of the angle *α* is controlled by

$$\dot{\alpha }=T(v_{\alpha }),\;\;{\dot{v}}_{\alpha }=f\left(U\right)\;T(v_{\alpha })-\nabla_{\alpha }\;U\left(\alpha ,\beta \right)\;,$$ (5)

where the objective function *U*(*α*, *β*) has the form of a mechanical potential, with ∇_*α*_ denoting the gradient with respect to *α*. Equivalent equations govern the time evolution of *β*. Eq (5) corresponds to a mechanical system with a potential *U* and a dissipation function *f*(*U*), for which the velocity *v*_*α*_ has been renormalized by *T*(*z*).

Mechanical systems with dissipation functions *f*(*U*) depending exclusively on the potential *U*, as in (5), can be considered on a general level as versatile prototype dynamical systems which exhibit, beside other, complex bifurcation cascades [25]. Several forms may be selected for the dissipation function *f*(*U*), as proposed further below. The system is adaptive [26], dispersing and taking up energy respectively for *f* < 0 and *f* > 0.

- In the dissipative stage, when *f*(*U*) < 0, the arm will follow a damped trajectory towards the next minimum of the potential *U* = *U*(*R*), that is towards the next object **m**_*i*_.
- For a dynamical dissipation function *f*(*U*), that is for a *f* = *f*(*U*) which depends functionally but not necessarily explicitly on the potential *U*, one can achieve that the state **r** ≈ **m**_*i*_ becomes progressively unstable, such that the arm eventually moves away from the object upon taking up energy after *f*(*U*) becomes positive.

The mechanical potential in (5) treats all targets **m**_*i*_ on an equal footing, the setup studied here.

### Dissipation function dynamics

The generic principle for selecting the dissipation function *f*(*U*) is that the system needs to be dissipative when far away from all objects **m**_*i*_, with the configuration **r** ≈ **m**_*i*_ becoming unstable once a specific target has been reached and followed for a certain time. Distinct ways to implement this principle are conceivable, here we study three possibilities.

- **Exponentially damped (ED)**. One may presume that the dissipation should become small far away from the objects, viz for large potentials *U*, as expressed by the ansatz
  $$\begin{array}{c} f\left(U\right)=f_{0}\text{exp}\left(-\mu \;U\right),\;\;\tau_{f}\;{\dot{f}}_{0}=E_{t}-U\;. \end{array}$$ (6)
  The prefactor *f*_0_ changes sign when the potential *U* stays below the reference energy *E*_*t*_ for a period comparable to *τ*_*f*_, viz when the end effector remains close to an object. Once *f*_0_ turns positive, the arm will start to move away from the current object **m**_*i*_.
- **Trailing potential (TP)**. In this setup the dissipation function is explicitly time dependent, with the evolution equation being determined by the trailing potential *U*_*T*_ = *U*_*T*_(*t*),
  $$\begin{array}{c} \tau_{f}\;\dot{f}=E_{t}-U_{T},\;\;\tau_{T}\;{\dot{U}}_{T}=U-U_{T}\;, \end{array}$$ (7)
  where the integration time scales are regulated by *τ*_*f*_ and *τ*_*T*_. The system is dissipative when *U*_*T*_ is large, taking up energy once it falls below the reference energy *E*_*t*_.
- **Adapting threshold (AT)**. One postulates that *f*(*U*) becomes positive when the potential *U* falls below a time dependent threshold *U*_*θ*_ = *U*_*θ*_(*t*):
  $$\begin{array}{c} f\left(U\right)=f_{0}\;(U_{\theta }-U)\text{exp}\left(-\mu U\right),\;\;\tau_{\theta }\;{\dot{U}}_{\theta }=E_{t}-U\;, \end{array}$$ (8)
  where *E*_*t*_ is a reference energy. The overall scale for *f*(*U*) is regulated by *f*_0_, with *τ*_*θ*_ determining the time needed for starting to take up energy, after the target has been reached dissipatively.

Further below we will present comparative results for the above three types of dissipation function dynamics, with in-detail investigations of robustness and other dynamical properties concentrating on ED.

### Moving objects

For the dynamics of the moving objects, the robot arm has to grab, we used two closely related algorithms.

- **Polar representation of the velocity (M-PV)**. In the first case the absolute velocity |*v*_*i*_| of an object **m**_*i*_ is drawn from an uniform distribution in [0, *a*], with the angle *φ*_*i*_ being drawn from [0, 2*π*].
- **Cartesian representation of the velocity (M-CV)**. In the second approach the Cartesian xy-components of **v**_*i*_ are drawn independently from an uniform distribution in [−*b*, *b*].

The resulting velocity **v**_*i*_ is applied in both cases for a time span *t*_*i*_ which is drawn uniformly from [0, *t*_*max*_]. The diffusion of the object is restricted in addition to a circular area of radius *r*_*area*_, reflecting at the boundary. We generally selected *r*_*area*_ to coincide with the reach of the robot arm. For the other parameters we took *a* = *b* = 0.001 and *t*_*max*_ = 10.

As the simulation results for M-PV and M-CV are very similar, we show in the following the ones for M-PV.

### Parameters

The overall length *L* = *l*_1_ + *l*_2_ of the arm is set to *L* = 2, with the lengths of the two segments being identical, *l*_1_ = *l*_2_ = 1. The parameters for the squashing function (4) for the distance are *κ*_*R*_ = 1 and $s_{R}=\sqrt{3/n}\;L/2$. For *n* = 3 moving objects we have hence *s*_*R*_ = *L*/2 = 1.

For the maximum of the potential *U*_*m*_ and for the reference energy *E*_*t*_ we used *U*_*m*_ = 17 and *E*_*t*_ = 0.05*U*_*m*_, respectively, with all other parameters being listed in Table 1. For the simulation a time step of *dt* = 0.01 has been used.

**Table 1:** Simulation parameters. The parameters *κ*_*v*_ and *s*_*v*_ entering the renormalization of the velocity of the mechanical system (5) have been adapted slightly for the three different dissipation function dynamics, ED, TP and AT. Listed are furthermore all parameters entering the respective defining Eqs (6), (7) and (8). Note that *μ* is given in units of 1/*U*_*m*_.

	*κ*_*v*_	*s*_*v*_	*μU*_*m*_	*τ*_*f*_	*τ*_*T*_	*τ*_*θ*_	*f*_0_
**ED**	2.8	1	25	1.2	•	•	•
**TP**	4.3	3	•	6.0	4.0	•	•
**AT**	4.0	2	34	•	•	1	0.5

## Results

**Fig 2:**
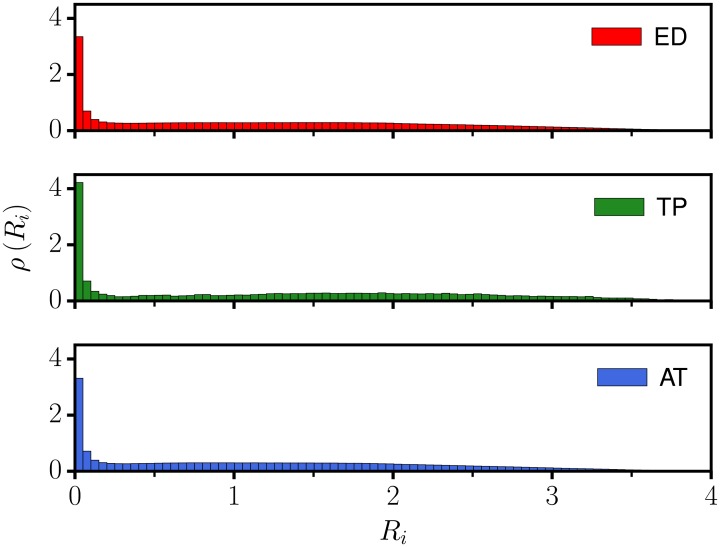
Distance statistics. The probability distribution *ρ*(*R*_*i*_) for he distance *R*_*i*_ between the end effector and a selected object *i*, as averaged over time. The targets are indistinguishable, which implies that *ρ*(*R*_*i*_) = *ρ*(*R*_*j*_) for all *i*, *j* ∈ [1, *n*], where *n* = 3 is the number of moving objects. Shown are the results for three different dissipation functions dynamics, ED (top, click for animation or see S1 Video), TP (middle, click for animation or see S2 Video), and AT (bottom, click for animation or see S3 Video), as defined respectively by (6), (7) and (8). The parameters are listed in Table 1.

For the parameters given in Table 1 we find transients in which the arm tends to stay close to a target it has approached. The flow in phase space is laminar when the arm is close to a target, accelerating however considerably once the dissipation function *f*(*U*) turns positive, compare (5) together with (6), (7) and (8). For a first understanding we present in Fig 2 the probability *ρ*(*R*_*i*_) to observe the distance *R*_*i*_ between the end effector and a given target *i*, see (3). With all *n* = 3 targets being equivalent, one has *ρ*(*R*_*i*_) = *ρ*(*R*_*j*_), for all *i*, *j* ∈ [1, *n*].

### Following vs. explorative phase

The distribution of the distance *R*_*i*_ presented in Fig 2 shows that the motion of the arm can be subdivided into a phase of small *R*_*i*_ and a phase of medium to large distances of all sizes, modulo fine details. That this is the case for three different types of dissipation function dynamics proves that the underlying generating principles is both robust and versatile. For the three variants considered here, (6), (7) and (8), the arm will start to take up energy whenever it did hover for a certain time close to a target, dissipating on the other side energy when far away.

**Fig 3:**
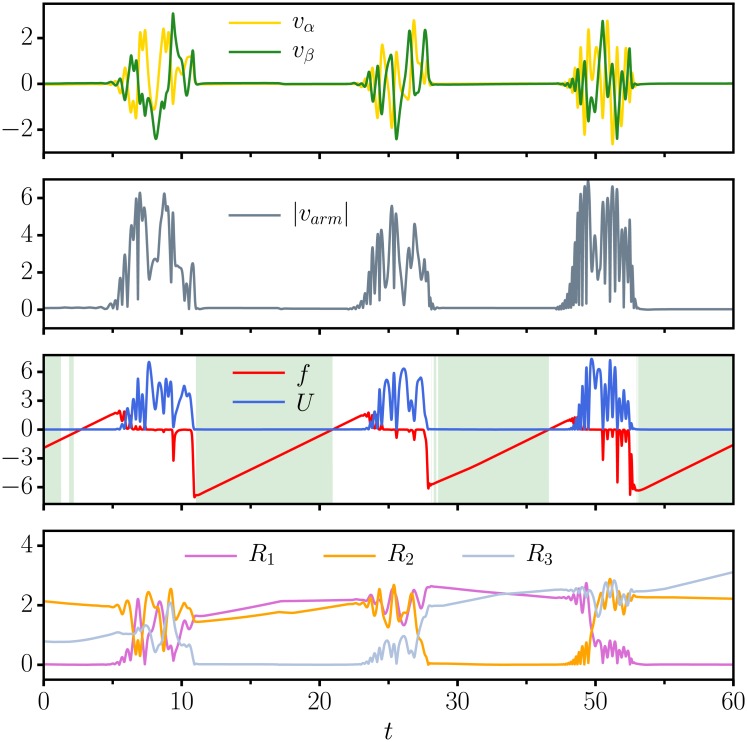
Time series for three moving objects. As a function of simulation time *t*, the evolution of key variables for the ED dissipation-function dynamics, compare (6). (top) The angular velocities *v*_*α*_ and *v*_*β*_. (second from top) The modulus |*v*_*arm*_| of velocity *v*_*arm*_ of the end effector. (second from bottom) The dissipation function *f* and the potential *U*, see (3), with the shading indicating that the criterion (9) is fulfilled. The separation of time scales characterizing the dynamics of *f*, for which a fast drop to negative values is followed by a slow recovery, drives the distinction between irregular searching phases and the laminar flow observed when the end-effector is close to a specific target. (bottom) The distances *R*_*i*_ to the *n* = 3 moving objects.

The evolution of key variables as a function of simulation time is presented in Fig 3. Shown are, for the ED dissipation function dynamics, the velocities *v*_*α*_, *v*_*β*_ and *v*_*arm*_, of the actuators and respectively of the arm, together with the evolution of the dissipation function *f*, of the potential *U*, and of the distances *R*_*i*_ between the hand of the arm and the individual objects.

One can distinguish in Fig 3 laminar ‘following phases’ and highly irregular ‘explorative phases’. Particularly evident is the driving role of the dissipation function, which remains negative for most of the smooth following phase. Visible is also a certain time lag between the crossing of *f* from negative to positive values, which results from the time the system needs to take up enough energy for the angular velocities *v*_*α*_ and *v*_*β*_, and the potential *U* to become visible.

### Robustness with respect to parameter changes

For a criterion that determines whether the end effector follows a given target we use

$$\begin{array}{c} U<E_{t},\;\;f\left(U\right)<0,\;\;\left|v_{arm}\right|<v_{tar}^{max}\;, \end{array}$$ (9)

which demands that the potential *U* is small with respect to the threshold energy *E*_*t*_ and that the system is momentarily dissipative, viz that the dissipation function *f*(*U*) is negative. The last term in (9) rules out coincidental crossings at high velocities, which occur when magnitude of the velocity *v*_*arm*_ of the end effector is larger than the maximal velocity $v_{tar}^{max}$ of the targets. With the dynamics of the targets being generated, as described, $v_{tar}^{max}$ is known. For practical applications it would be in any case sufficient to use an empirical estimate for $v_{tar}^{max}$.

Using the criterion (9), one can define a probability *P*_*close*_ that measures the relative fraction of time the arm follows a target, with following and the exploration being the two dominant states of the system, as evident from Fig 3.

**Fig 4:**
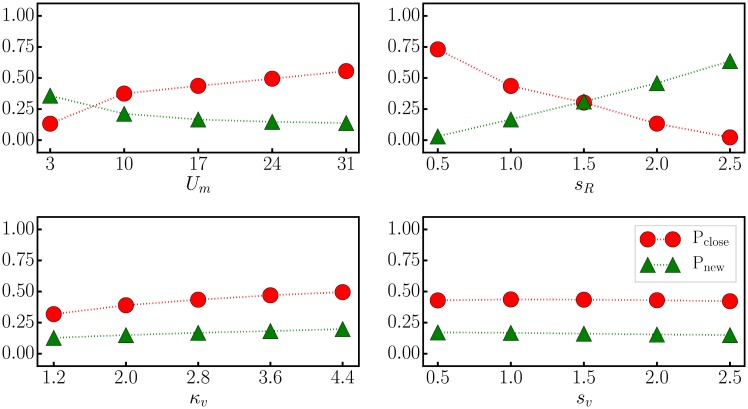
Parameter sweep. For the ED dissipation function dynamics, the probability *P*_*close*_ for the arm to be close to one of the *n* = 3 targets (red circles), as defined by (9), and *P*_*new*_, which measures the chance that two targets approached one after the another are different (green triangles). With respect to the reference values *U*_*m*_ = 17, *s*_*R*_ = *L*/2 = 1, *κ*_*v*_ = 2.8 and *s*_*v*_ = 1, the values of the parameters have been changed individually.

In Fig 4 we present for the ED dissipation function dynamics the numerical result for *P*_*close*_. Starting from the reference set of parameters *U*_*m*_ = 17, *s*_*R*_ = *L*/2 = 1, *κ*_*v*_ = 2.8 and *s*_*v*_ = 1, compare also Table 1, the parameters have been modified one by one and the probability for the arm to follow a target evaluated. Also included in Fig 4 is the probability *P*_*new*_, namely that two targets approached successively differ.

- The probability *P*_*close*_ for the arm to be in the following phase increases monotonically with the strength *U*_*m*_ of the potential, an intuitive result. *P*_*new*_ decreases conversely, with the reason being that a larger *U*_*m*_ makes it more difficult to escape the local potential well.
- Increasing the characteristic length *s*_*R*_ for the distance between the arm and a target, which enters the squashing function (4), decreases *P*_*close*_ dramatically. This is because the local potential wells attracting the end actuator to a target in first place tend to disappear for large *s*_*R*_. *P*_*new*_ increases on the other side.
- The squashing parameters *κ*_*v*_ and *s*_*v*_ for the velocity of the actuators can be changed considerable without affecting either *P*_*close*_ or *P*_*new*_, implying that the system is robust with respect to both *κ*_*v*_ and *s*_*v*_.

**Fig 5:**
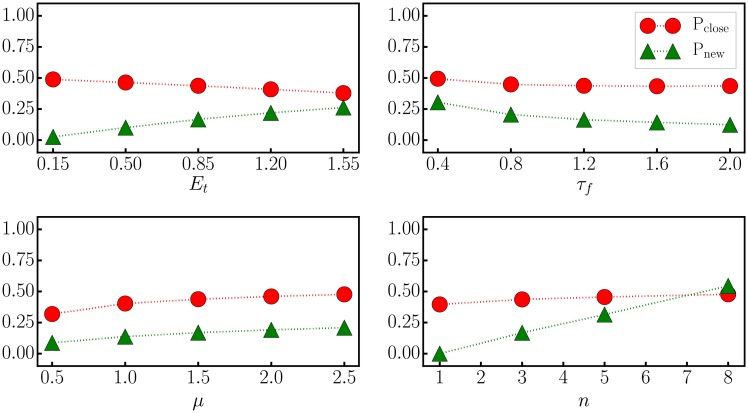
Robustness of the dissipation function dynamics. For the ED dissipation function dynamics, the probability *P*_*close*_ for the arm to be close to a target (red circles), as defined by (9), and *P*_*new*_, which measures the chance that two targets approached one after the another are different (green triangles). With respect to the reference values *E*_*t*_ = 0.05*U*_*m*_ = 0.85, *μ* = 25/*U*_*m*_ = 1.47 *τ*_*f*_ = 1.2, the values of the parameters have been changed individually for *n* = 3. Also included are the values of *P*_*close*_ and *P*_*new*_ upon changing the number *n* of targets. Here $s_{R}=\sqrt{3/n}L/2$.

The data shown in Fig 4 describes the influence of global parameters. In Fig 5 we present for completeness the effect of changing the parameters *E*_*t*_, *μ* and *τ*_*f*_ of the ED dissipation function dynamics, see (6). We find the generating principle to be robust, viz that the dependency of *P*_*close*_ and *P*_*new*_ on *E*_*t*_, *μ* and *τ*_*f*_ is moderate.

Also included in Fig 5 are the values of *P*_*close*_ and *P*_*new*_ obtained upon changing the number *n* of targets. One observes that the relative fraction of time *P*_*close*_ the arm spends close to a target remains flat. For *n* = 1 the probability to change targets vanishes, as it must, becoming on the other side substantial for large numbers of targets *n*.

The here presented sequential task-switching behavior, generated by the prototype dynamical system (5) does not rely on the particular choice of the generalized dissipation function dynamics. As demonstrated by Fig 2, similar distance distributions *ρ*(*R*_*i*_) may result from very different dissipation function implementations. This is also reflected by the fraction of time spent with following and the probability of switching targets, *P*_*close*_ = 0.44/0.69/0.44 and *P*_*new*_ = 0.17/0.07/0.14, when comparing the dissipation functions ED/TP/AT see Eqs (6), (7) and (8) respectively, for the parameters given in Table 1.

### Robustness with respect to target properties

**Fig 6:**
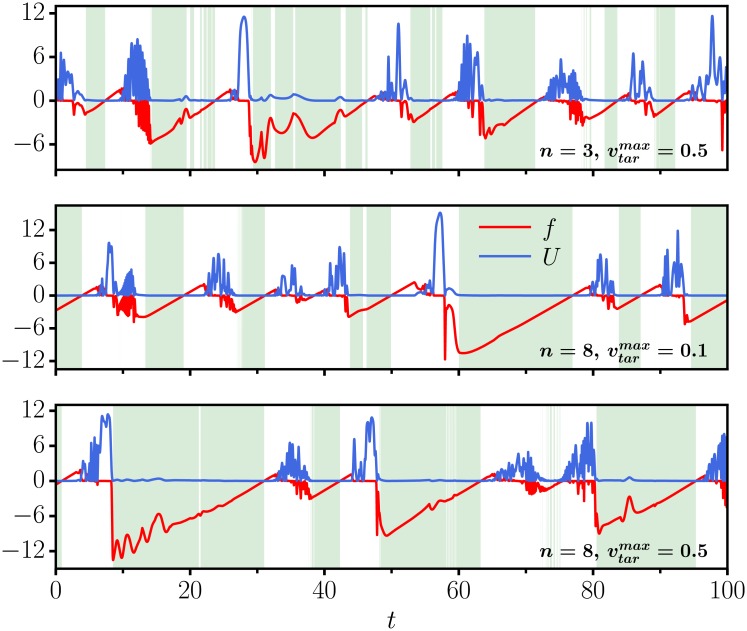
Variable object characteristics. As a function of simulation time *t*, the evolution of the dissipation function *f* (red) and of the potential *U* (blue) for the ED dissipation-function dynamics. The shaded regions indicate that the criterion (9) for the arm to be in the following phase is fulfilled. (top) For *n* = 3 objects for which the maximal velocity is 0.5, viz five times larger than in Fig 3 (click for animation or see S4 Video). (middle) For *n* = 8 objects with a maximal velocity 0.1 (click for animation or see S5 Video). (bottom) For *n* = 8 objects with a maximal velocity 0.5 (click for animation or see S6 Video).

It is clear that the arm would not be able to follow a target if the maximal velocity $v_{tar}^{max}$ is too large. We find, however, that the here proposed generating principle works for a substantial range of $v_{tar}^{max}$. For the ED dissipation function dynamics we present in Fig 6 the time series of the dissipation function and of the potential both for the case of $v_{tar}^{max}=0.1$, as used hitherto, and for $v_{tar}^{max}=0.5$. We find that only details of the overall dynamics change. This holds also when increasing the number of moving objects from *n* = 3 to *n* = 8.

### A single non-moving target

From the dynamical system perspective it is of interest to investigate the case of a single stationary target. With noise being absent, the system is deterministic.

**Fig 7:**
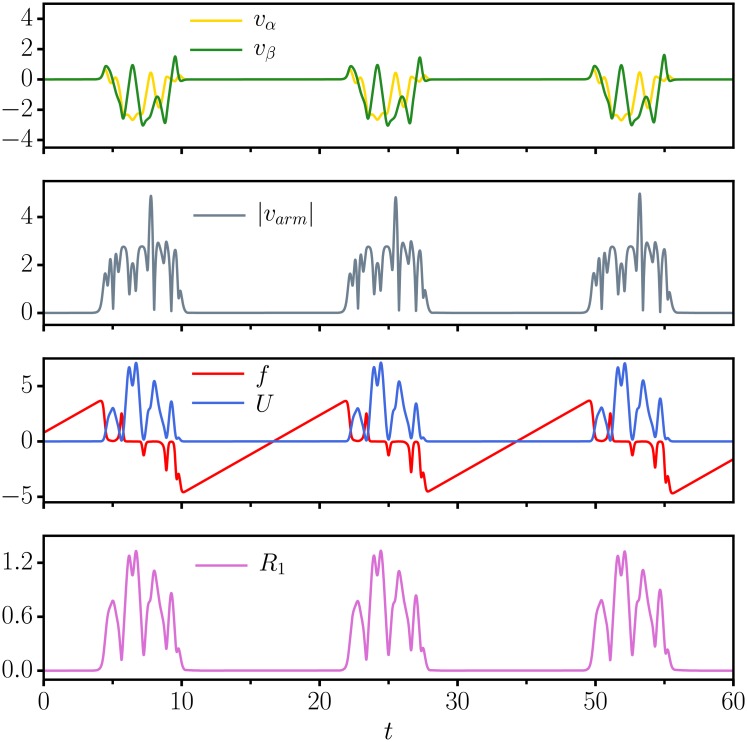
A single non-moving object. As a function of simulation time *t*, the evolution of key variables for the ED dissipation-function dynamics, as for Fig 3, but here for a single non-moving object located at (3/8, 3/8)*L*. The system is fully deterministic, with the robot arm settling into a limit cycle. The criterion (9) for the arm to be close to the object is not applicable, as $v_{tar}^{max}=0$.

- **Fixpoints**. In case of a purely dissipative dynamics, with *f*(*U*) = *f*_0_ < 0, the system disposes of two stable fixpoints, defined by vanshing angular velocities *v*_*α*_, *v*_*β*_ → 0, that correspond to a right- and respectively to a left bend.
- **Limit cycle attractors**. With the dynamical dissipation function ED, it is evident that the robot arm settles into a limit cycle in which the destabilized fixpoints are revisited, see Fig 7. There exist, hence, multiple symmetry related limit cycles even for a single resting target (only one of them is shown).

Therefore, in the presence of multiple fixed targets, several different activity sequences may be generated, even for the same starting position **r**(0) of the arm, viz for different initial conditions of the internal variables.

## Discussion

Action switching in embodied agents may be guided by fitness considerations, f.i. when the task is to collect a series of different food sources [27]. Typically, the action selected at a given time will be then the one with the most pressing need. We have followed here a different approach, examining an overarching generation principle and not the generation of action sequences driven by an utility optimization that is local in time.

### The stationarity principle

The question how to decide in which action to engage has been termed the motivational problem [28]. The utility of many activities, like foraging, socializing and resting, that are regularly repeated, address distinct needs, which implies that they cannot be lumped together into an overarching utility function. In terms of multi-objective optimization [24] the agent must dedicate time to a range of activities, with the constraint that the resulting distribution of utilities remains within a given range. This constraint may be expressed as a stationarity principle, namely that the statistical properties of the time series of activities should become stationary for extended time spans.

The result presented here for the self-organized robot arm can be viewed as an implementation of the stationarity principle. With the dynamics being irregular, viz chaotic, in the explorative phase, the exact sequence of objects followed is not pre-determined. The long term statistics, such as the distance distribution presented in Fig 2, is however stationary.

The stationarity principle is a guiding principle that can be used in various settings. Statistical learning, e.g. of receptive fields [29], is characterized by statistically stationary sensory inputs, with learning continuing until the statistics of the output activity becomes also stationary [30]. It has been shown, that one can use the Fisher information of the neural firing rate to encode the stationarity principle [31] and that one obtains Hebbian learning when minimizing the Fisher information, viz when the stationarity condition is enforced.

### Transient-state dynamics

With the agent being formulated in term of a mechanical system, see Eq (5), one can abstract from the behavioral level and describe the robot arm within dynamical system theory [26]. The striking alternation of dynamical states, as visible in Fig 3, can be interpreted in this context as an example of transient-state dynamics [32]. The following phase corresponds on a dynamical level to a transient attractor that becomes unstable on an extended time scale, namely when the dissipation function turns positive.

The here discussed mechanism, the coupling of an attracting state to a slow variable, is the core route for generating transient-state dynamics in general [33], with the flow being laminar during the transient dynamics, and irregular during the transition periods. We note that transient-state dynamics may be viewed as a form of metastability, which may arise either from the brain dynamics as such [34], or from sensorimotor couplings in response to tasks demanding behavioral flexibility [35].

### Distinguishable vs. non-distinguishable targets

It would be possible to introduce a bias *b*_*i*_ = *b*_*i*_(*t*) that allows to differentiate between distinct objects. In this case one would work with the generalized Euclidean distance

$$\begin{array}{c} R_{i}\;\to \;\sqrt{R_{i}^{2}+b_{i}^{2}}\;. \end{array}$$ (10)

instead of (3), for which the bias *b*_*i*_ encodes the depth of the potential, and with this indirectly also the relative importance of the respective object. For an appropriate evolution equation for *b*_*i*_(*t*), the respective target would become repelling once the end effector of the robot has reached it. Two routes on how the dynamical system (5) induces an autonomously generated sequence of behaviors are hence possible.

- **Distinguishable targets**. One works with a constant dissipation function, *f*(*U*) → *f*_0_, with every object being characterized by a time-dependent attribute, namely *b*_*i*_ = *b*_*i*_(*t*).
- **Indistinguishable targets**. When all *b*_*i*_ ≡ 0 there is no variable distinguishing the individual objects. The sequence of behaviors is then a consequence of dynamical instabilities resulting from the dynamics of the dissipation function.

In this study we concentrate on the second case as the basic generative mechanism, noting that the resulting residence times, viz when **r** ≈ **m**_*i*_, could be fine-tuned in a second step by allowing the *b*_*i*_ to be weakly time dependent. This protocol is left for future studies.

## Conclusion

One of the biggest challenges in the design of controllers for autonomous agents is the combination of different goal oriented behaviors into a series of self-organized activities [36]. Here, we investigated how such a higher order controller may be constructed within a dynamical systems framework, by adapting a recently introduced versatile prototype system [25] to the problem of an object-following arm. By introducing a model with a dynamically changing generalized dissipation function we provide a proof of concept demonstration of how target following can be turned into a sequential task switching behavior in terms of transient-state dynamics [32].

Within this framework the goal oriented activities are represented by a target-following behavior of a simulated arm, while the switching dynamics between targets corresponds to an explorative phase upon getting bored of the respective task.

Such a self-organized behavior can be generated both at the level of motion primitives, in case of robotic locomotion [10], and on the level of action selection [27], as demonstrated here. The resulting behavior is robust within a wide range of parameters, as it does not require precise fine tuning, which simplifies the selection of an adequate parameter set with, e.g., machine learning techniques. Being based on self-organized attractors in the overarching phase space of agent and environment, the sensorimotor loop, our approach is resistant to external noise, retaining at the same time the flexibility to adapt to the environment or to interact with other agents [15].

The proposed framework can be generalized to produce series of activities with a well-defined order or a given multi-modal probability distribution by modulating the Euclidean distance as a function of the actual importance of the respective task – a research direction left for future studies.

## Supporting information

S1 Video Video for ED dissipation dynamics. Illustrating video for Fig 2. For *n* = 3 moving objects, a maximal object velocity of 0.1 and the ED dissipation dynamics, as defined by (6).
(MP4)

S2 Video Video for TP dissipation dynamics. Illustrating video for Fig 2. For *n* = 3 moving objects, a maximal object velocity of 0.1 and the TP dissipation dynamics, as defined by (7).
(MP4)

S3 Video Video for AT dissipation dynamics. Illustrating video for Fig 2. For *n* = 3 moving objects, a maximal object velocity of 0.1 and the AT dissipation dynamics, as defined by (8).
(MP4)

S4 Video Video for ED dissipation dynamics. Illustrating video for Fig 6. For *n* = 3 moving objects, a maximal object velocity of 0.5 and the ED dissipation dynamics, as defined by (6).
(MP4)

S5 Video Video for ED dissipation dynamics. Illustrating video for Fig 6. For *n* = 8 moving objects, a maximal object velocity of 0.1 and the ED dissipation dynamics, as defined by (6).
(MP4)

S6 Video Video for ED dissipation dynamics. Illustrating video for Fig 6. For *n* = 8 moving objects, a maximal object velocity of 0.5 and the ED dissipation dynamics, as defined by (6).
(MP4)
